# Potential Role of Antioxidants as Adjunctive Therapy in Chagas Disease

**DOI:** 10.1155/2020/9081813

**Published:** 2020-03-26

**Authors:** Juana P. Sánchez-Villamil, Paula K. Bautista-Niño, Norma C. Serrano, Melvin Y. Rincon, Nisha J. Garg

**Affiliations:** ^1^Translational Biomedical Research Group, Centro de Investigaciones, Fundación Cardiovascular de Colombia, Santander, Colombia; ^2^Faculty of Basic Sciences, Universidad Antonio Nariño, Santander, Colombia; ^3^Department of Microbiology and Immunology, Institute for Human Infections and Immunity, University of Texas Medical Branch, Galveston, Texas, USA

## Abstract

Chagas disease (CD) is one of the most important neglected tropical diseases in the American continent. Host-derived nitroxidative stress in response to *Trypanosoma cruzi* infection can induce tissue damage contributing to the progression of Chagas disease. Antioxidant supplementation has been suggested as adjuvant therapy to current treatment. In this article, we synthesize and discuss the current evidence regarding the use of antioxidants as adjunctive compounds to fight harmful reactive oxygen species and lower the tissue oxidative damage during progression of chronic Chagas disease. Several antioxidants evaluated in recent studies have shown potential benefits for the control of oxidative stress in the host's tissues. Melatonin, resveratrol, the combination of vitamin C/vitamin E (vitC/vitE) or curcumin/benznidazole, and mitochondria-targeted antioxidants seem to be beneficial in reducing plasma and cardiac levels of lipid peroxidation products. Nevertheless, further research is needed to validate beneficial effects of antioxidant therapies in Chagas disease.

## 1. Introduction

Chagas disease (CD), caused by *Trypanosoma cruzi*, belongs to the group of neglected tropical diseases that affects more than 1 billion of the poorest and most marginalized people in the world [1]. It is estimated that 6-7 million people are infected with *T. cruzi*, ~30,000 new cases of *T. cruzi* infection emerge each year, and CD accounts for >12,000 deaths *per* year (World Health Organization (WHO) Epidemiology, http://www.who.int/chagas/epidemiology/en/). *T. cruzi* infection and CD are endemic in South America, Central America, and Mexico. The natural cycle of *T. cruzi* transmission evidenced with the detection of a high rate of infection in dogs [2, 3] and autochthonous cases of CD in humans [4] is also documented in the Southern USA. Since 1980s, due to the migration of infected woman of childbearing age to nonendemic regions of the world who transmit the infection to their babies, the incidences of CD have increased, transforming it into a new worldwide public health challenge [5].

Upon infection, acute blood parasitemia can be detected for approximately 60 days by various diagnostic methods (discussed in [6]). Most infected individuals develop potent immune response to control *T. cruzi* infection; however, the parasite persists at low levels in the host, and a vast majority of infected individuals develop no organ dysfunction during their life. However, up to 1/3^rd^ of the infection cases progress into the clinical form of the disease that mainly develops with the pathological involvement of the heart, though the megaesophagus and megacolon may also be noted [7, 8]. Chagas cardiomyopathy is presented with a wide variety of manifestations including arrhythmias, apical aneurysm, left ventricular systolic dysfunction, thrombotic events, dilated cardiomyopathy, and terminal heart failure leading to patients' death [9].

Two drugs, benznidazole and nifurtimox, are currently available for the treatment of patients diagnosed early after *T. cruzi* infection. International guidelines recommend that acute infection cases (all ages) and children up to 14 years old should be treated with antiparasitic drug therapies [10]. In the US, the Food and Drug Administration agency has approved benznidazole for use in children 2–12 years of age [11]. Though the mechanism of action is not completely understood, it is suggested that the activated benznidazole and nifurtimox (and their metabolites) bind to and block the parasites' antioxidant availability [12, 13] and generate DNA-toxic glyoxal adducts [14] causing oxidative damage to the parasite [15, 16]. It is important to note that benznidazole and nifurtimox have limited efficacy in the chronic disease phase [17] when adult patients exhibit several significant side effects [17], and these drugs are not recommended for pregnant women (reviewed in [18, 19]). Thus, there is an urgent need for new drugs to control pathogen and pathogen-induced pathological events in CD [20].

The pathology of Chagas disease is complex, with several parasitic and host determinants having critical and major roles. Parasite virulence and genetic susceptibility of the host result in varying disease outcomes. In general, it is believed that the low-grade parasites contribute to heart damage through inducing inflammation, fibrosis, and oxidative injuries, leading to disruption of myofibrils, myocyte necrosis, microvascular and autonomic dysfunction, and cardiac hypertrophy and fibrosis. Depending on the extent of these processes, varied outcomes of infection ranging from no disease to cardiac damage, remodeling, and heart failure and related clinical sequelae, such as stroke, may culminate in the patient. Readers are directed to an excellent recent review for additional details on the pathology and pathogenesis of Chagas heart disease [9].

## 2. Antioxidant/Oxidant Imbalance in Chagas Disease

Antioxidant/oxidant imbalance is considered a main factor associated with CD progression. With regard to elicitation of oxidative stress, two major sources are identified. Studies in mice and humans show that innate and adaptive immune responses should control the parasite through the production of reactive oxygen species (ROS)/reactive nitrogen species (RNS), proinflammatory T_H_1 cytokines, trypanolytic antibodies, and cytotoxic T lymphocytes' activity (reviewed in [6, 9]). Macrophages and other innate immune cells that offer the first line of defense respond to *T. cruzi* infection through an immediate increase in the expression of proinflammatory cytokines followed by subpar production of superoxide (O_2_^·−^) and nitric oxide (NO) by NADPH oxidase (NOX2) and inducible nitric oxide synthase (iNOS), respectively [21–23]. The reaction of O_2_^·−^ with NO produces a strong cytotoxic oxidant peroxynitrite (ONOO^−^). While some studies have indicated that peroxynitrite and other powerful cytotoxic effectors produced by macrophages are essential for killing parasites [24], others have indicated that the macrophage oxidative environment acts as an enhancer of infection [25]. Regardless, parasites are able to persist in this oxidative environment and the ONOO^−^ radical can exert cytotoxic effects on the host cells [24].

Nonimmune cells, e.g., cardiac myocytes, have also been shown to respond to *T. cruzi* infection through ROS production. Indeed, several studies have identified mitochondria as the main source of the ROS in *T. cruzi*-infected cardiomyocytes [26, 27]. Typically, perturbation of membrane potential or loss of structural integrity of the mitochondrial membranes [26] adversely affects the activity of the electron transport chain and results in increased mitochondrial ROS (mtROS) production [28, 29]. Specifically, activities of the respiratory complex I and complex III were compromised, and electron leakage to O_2_ and O_2_^·−^ production occurred at the Q1 semiubiquinone of complex III in infected cardiomyocytes and CD hearts [26, 30]. Mitochondrial dysfunction has been well documented by us and other researchers in cardiomyocytes infected by *T. cruzi* and in the myocardium of chronically infected animals [31, 32] and clinically symptomatic CD patients [28, 33–35]. *In vivo* studies in rodents also showed that mitochondrial defects and high mtROS levels persist in the chronic phase of infection [36, 37].

A network of enzymatic and nonenzymatic antioxidants control oxidative stress. However, several studies have indicated that the increase in mtROS production correlated with a decline in the expression and activity of the mitochondrial antioxidant enzyme Mn^+2^ superoxide dismutase (MnSOD) and a decline in the cytosolic glutathione peroxidase (GPx) activity and GSH content in the myocardium of chronically infected animals and in Chagas patients [28, 34, 35, 38, 39]. NFE2L2 (also called Nrf2) is a transcription factor that regulates the expression of antioxidant proteins. A recent study showed that NFE2L2 expression, nuclear translocation, and binding to cis-acting DNA regulatory antioxidant response elements (ARE) were significantly decreased and associated with a decline in antioxidant levels (e.g., *γ*GCS, HO1, GCLM) in cardiac myocytes and the myocardium of mice infected with *T. cruzi*. Overexpression of MnSOD in cardiac myocytes preserved the NFE2L2 transcriptional activity and antioxidant/oxidant balance, and MnSOD^tg^ mice also preserved the cardiac structure and function [40]. This study provides evidence that mtROS inhibition of the NFE2L2/ARE pathway constitutes a key mechanism in signaling the fibrotic gene expression and evolution of chronic Chagas cardiomyopathy.

In summary, a balance between the levels of ROS that are capable of inducing parasite damage and the antioxidant machinery that the host requires to detoxify and keep a safe environment for cells exposed to infection is fundamental. However, the host antioxidant response is exhausted during progression of Chagas disease [36, 38, 40, 41] and a lack of appropriate antioxidant and repair response results in self-perpetuating mitochondrial dysfunction and ROS production in the heart (reviewed in [9, 42]). The increased and sustained ROS production can signal the fibrotic gene expression and contribute to evolution of chronic cardiomyopathy [40, 43]. ROS can also elicit a consequent antioxidant depletion and immunological response that causes persistent inflammation and oxidative damage of proteins, lipids, and DNA leading to the pathological tissue manifestations in CD [28, 35, 44].

In these circumstances, it is necessary to review the current and new emerging evidence about antioxidant administration in CD. Several antioxidants have been studied as therapeutics for diverse pathologies in preclinical models [45]. Accordingly, we proceeded to (i) synthesize the published evidence on the use of antioxidants in CD, including experimental and preclinical research, (ii) describe the main characteristics of the published studies to shape the directions for future research, and (iii) discuss the potential usefulness of antioxidants as complementary or adjunct therapy with antiparasitic drugs for control of oxidative tissue damage and Chagas cardiomyopathy.

## 3. Antioxidant Effects in Experimental Models of Chagas Disease

Several antioxidant compounds have been examined as an option for multiple diseases. Their therapeutic goal is to prevent, attenuate, or block the oxidative damage of the host cells [46, 47]. In the context of CD, novel approaches are envisioned to reduce the oxidative damage in the host while maintaining the effectiveness of the antitrypanosomal agents. We appraised the published literature focusing on evaluation of the markers of oxidative stress and benefits of antioxidant treatment in CD. Except for some studies testing vitamin C/vitamin E, all other studies have not evaluated the effect of antioxidants' treatment on parasite levels or parasite persistence. Instead, most of the studies (fifteen total) were conducted in experimental animal models and primarily focused on measuring the oxidative stress levels (Table 1). The major characteristics of these studies are as follows: The potential effect of the antioxidants in terms of control of oxidative stress has been assessed *in vivo* using murine models infected with *T. cruzi*, in both acute and chronic infection. Swiss outbred mice were used in 53.3% of the studies. The animal's age for experiments ranged between 3 and 12 weeks old. Only one study by Novaes et al. [48] used 12-month-old mice. In 82% of the studies, male animals were used without a clear reference to gender exclusion parameter. However, some researchers have reported an association between males, ROS levels, and increased severity of myocardial fibrosis associated with CD [49], thus justifying the use of males for a majority of published studies examining the antioxidants' efficacy in Chagas disease. Challenge infection was performed in all the studies by intraperitoneal inoculation of *T. cruzi*. Infection was defined as acute up to 60 days after inoculation and as chronic from day 120 after inoculation. The most frequently used parasite strain was *T. cruzi Y* strain (*n* = 5 studies; 33.3%). Others used strains included *QM1*, *QM2*, *Sylvio X10/4*, *Brazil*, *Colombian*, and *Ninoa*. The number of parasites used for challenge infection was variable, ranging from 1 × 10^1^ to 1 × 10^5^ blood trypomastigotes, that was mostly related to virulence of the parasite isolates but unrelated to the development of the acute or chronic phase.

The antioxidant compounds used in these studies included phenyl-*α*-tert-butyl nitrone (PBN) [50, 51], carvedilol, vitamin E (vitE) and/or vitamin C (vitC) [52, 53], melatonin [54], curcumin [55], resveratrol, and astaxanthin [56]. Vitamin C was the most commonly used antioxidant, evaluated in five of the published studies. Three of the antioxidant compounds, vitC, curcumin, and PBN, were also evaluated in combination with benznidazole. Only astaxanthin was evaluated in combination with nifurtimox [56] (Table 1). The oral route of administration of the antioxidant compounds was used in >80% the studies. The oxidative stress markers commonly measured as an indicator of tissue injury included MDA, protein carbonyls (PCN), lipid hydroperoxides (LPO), and nitrites. The response of the endogenous antioxidant system was determined by evaluating the concentration of glutathione, and activities of catalase, SOD, GPx, and other antioxidant enzymes in the blood or cardiac tissues. We discuss in detail the effect of the different antioxidant treatment strategies in CD below.

### 3.1. Vitamins C and E

Ascorbic acid or vitC is one of the most potent antioxidant agents, but at the same time, it acts as a prooxidant [57]. VitC is also the most frequently studied antioxidant in CD (Table 1). Most studies used male Swiss mice, but they differed in experimental conditions like *T. cruzi* strain, parasitic or antioxidant dose, or the treatment time period. Two studies evaluated the effects of vitC and vitE during the acute phase, two during the chronic phase, and one study in both phases of experimental CD. When used in the acute parasitemia phase, vitC treatment was given during 15 to 60 days postinfection (Table 1).

Providello et al. tested vitC with or without benznidazole in acutely infected rodents [58]. The authors showed that treatment with vitC in combination with benznidazole rather than alone resulted in a significant decline in thiobarbituric acid reactive substances (TBARS) in the cardiac tissue of infected mice and decreased ROS generation by host macrophages [58]. Likewise, Puente et al. [59] reported that vitC exerts a prooxidant effect on the parasite, while it had an antioxidant effect on the host [59]. These authors noted that treatment with vitC did not affect the antiparasitic activity of benznidazole; however, vitC decreased the cytotoxicity of benznidazole on host cells and vitC-treated mice exhibited no weight loss and no mortality in response to acute *T. cruzi* infection [59].

Tieghi et al. evaluated the effect of vitC and vitE (individually and in combination) in acutely and chronically infected Swiss mice [53]. The authors noted that supplementation with vitC, vitE, or both had no effect on parasitemia levels. VitC treatment improved the plasma antioxidant capacity and glutathione levels in acutely infected mice, and the lipid peroxidation levels in the plasma and heart tissue of chronically infected mice. Further, vitE showed a synergistic effect with vitC in increasing GSH levels in the acute phase and reducing the plasmatic lipoperoxidation during the chronic phase. In contrast, treatment with vitE only had a prooxidant effect evidenced by an increase in lipid peroxidation in the skeletal muscle of acutely infected mice and no antioxidant effect in chronic Chagas mice. In another study, Novaes et al. [48] used a similar vitE dose as reported by Tieghi et al. [53] and showed reduction in lipid peroxidation in the cardiac muscle of vitE-treated infected mice, though these authors also noted no increase in the antioxidant enzyme activities. Others showed that vitC had a prooxidant effect in Chagas mice, though this seemed to be a dose-dependent effect; treatment with 60 mg vitC was not oxidative, while treatment with 500 mg of vitC per day led to increased total peroxide and TBARS at 60 and 180 days [52, 60].

In summary, therapeutic administration of vitC or vitE alone was unable to protect against oxidative stress in both acute and chronic phases of CD. Interestingly, vitC and vitE have a prooxidant effect by increasing ROS production, which can be harmful to the host due to the increased severity of tissue lesions but beneficial when used in cases of low parasitemia. A reduction in the parasitic burden occurs because vitC also stimulates NO production [52], which has been associated with a trypanocidal effect [61, 62]. VitC, in combination with vitE, exerted slight advantages in controlling oxidative damage in Chagas mice.

### 3.2. Phenyl-alfa-tert-butyl Nitrone (PBN)

PBN is a nitrone-based antioxidant that scavenges free radical species and inhibits free radical generation [63]. The effects of PBN in CD have been reported in rats during the acute and chronic phases of infection using an oral administration of 1.3 mM of PBN for three weeks [51, 64] and in mice during the acute phase with injection delivery of 50 mg/kg of PBN [50]. Treatment with PBN controlled the pathologic oxidative tissue injury and preserved mitochondrial respiratory chain function in both acutely and chronically infected rodents. Wen and Garg [64] also showed that PBN restored the differential expression of 65% of the disease-associated proteins to the normal level and prevented the development of oxidative adducts on plasma proteins in chronically infected rats [51, 64]. Importantly, PBN alone or in combination with benznidazole (but not benznidazole alone) preserved the left ventricular function that otherwise was significantly compromised in chronically infected Chagas rats [51, 64].

### 3.3. Mitochondria-Targeted Antioxidants

Currently, the application of mitochondria-targeted therapies for parasitic diseases has focused on studying the mitochondrial function in *Leishmania* and other trypanosome parasites [65], owing to the fact that their mitochondria are different from human mitochondria. However, mitochondria-based therapies designed to improve mitochondrial health of the patients are also needed. This is because mitochondrial dysfunction contributes to the pathology of Chagas cardiomyopathy in the same way as many other disorders like neurodegeneration, metabolic disease, heart failure, and ischemia-reperfusion injury, for which several therapeutic strategies to restore mitochondrial function are emerging in recent years [66]. Encouraging results have been obtained from treatments targeting mitochondrial bioenergetics for the improvement of heart function in animal models of heart failure [67]. These same therapies can be promising alternatives to preserve mitochondrial bioenergetics and as a consequent oxidant/antioxidant balance in Chagas cardiomyopathy.

There are several mitochondria-targeted antioxidants, and the more notable among them are mitoquinone (MitoQ), MitoTEMPO, Tempol, and Mn-porphyrin. Only one mitochondria-targeted antioxidant has been studied focusing on the control of mitochondrial dysfunction and oxidative pathology in CD and it is described below:

#### 3.3.1. Tempol (SOD-Mimetic)

Tempol is a superoxide mimetic agent that has been evaluated for treatment of many diseases related to mitochondrial dysfunction and oxidative stress [68]. Vilar-Pereira et al. have evaluated the protective action of Tempol in CD, although this was used to validate findings with resveratrol treatment [69]. Chagas mice chronically infected with the Colombian strain of *T. cruzi* were mice treated with 100 mg/kg of Tempol (SOD2 mimetic) or 500 mg/kg of metformin that were administrated daily by gavage for 30 days, beginning 60 days postinfection. The authors showed that Tempol decreased lipid peroxidation and improved heart function in Chagas mice. Likewise, metformin (agonist of AMP-activated protein kinase, AMPK) that is used for the treatment of type 2 diabetes and is shown to inhibit mitochondrial complex I, reduce ROS, and modulate other cellular mechanisms associated with extension of lifespan [70] also reduced lipid peroxidation in Chagas mice [69].

### 3.4. Resveratrol

Resveratrol is a phenol found in many plants and fruits with a demonstrated role in the control of tissue damage in degenerative diseases and cancer. However, its major therapeutic role as a cardiovascular protector is documented in the heart failure of different etiologies [71]. Resveratrol is shown to have anti-inflammatory, antimicrobial, and antioxidant properties [72] and had inducing effects on endothelial NO synthase (eNOS) [73] and various antioxidant enzymes [74], while increased scavenging of superoxide, hydroxyl, and peroxyl radicals was also noted [74].

Vilar-Pereira et al. [69] showed that treatment of chronically infected mice with resveratrol for 30 days normalized the Mn-superoxide (SOD2) levels and significantly decreased the lipid peroxidation in the hearts of Chagas mice. Reducing ROS-induced oxidative adducts was enough to improve the heart function, although histological structure of the heart showed little-to-no improvement and there was no correlation with changes in PGC1-*α* expression or ATP levels that are the molecular regulators of oxidative metabolism. Authors noted that Chagas mice treated with resveratrol exhibited increase in the activation of the AMPK pathway required for maintaining the cellular energy homeostasis [75].

### 3.5. Melatonin

Melatonin is a potent antioxidant known to neutralize hydroxyl and peroxyl radicals [76]. Melatonin is shown to be effective in reducing oxidative stress in many pathological conditions [77]. Brazao et al. [54] showed that the oral administration of melatonin (5 mg/kg of body weight) for 60 days during chronic *T. cruzi* infection arrested the plasmatic lipid peroxidation. Authors showed lower levels of NO production in melatonin-treated animals and proposed that melatonin controlled the NO-dependent formation of cytotoxic ONOO^−^ in Chagas mice [54]. Because NO has a trypanocidal effect [61], it is possible that melatonin may also control the parasite burden; however, this was not examined in detail in this study [54].

### 3.6. Curcumin

Curcumin is a natural phenolic antioxidant and free radical scavenger that reduces the release of superoxide radicals, hydrogen peroxide, and nitric oxide in immune cells [78] and inhibits lipid peroxidation [79]. Published literature suggests that curcumin modulates multiple signaling molecules, transcription factors, and protein kinases and protein reductases linked to cardiovascular, metabolic, and other chronic diseases. [80]. Curcumin is suggested to confer therapeutic benefits, either alone or in combination with other agents, through intrinsic antioxidant, anti-inflammatory, anticancer, antiatherosclerotic, and antiaging properties [81, 82]. Curcumin has been studied in CD as well as in other parasitic diseases, focused mainly on its immunomodulatory and antiparasitic action because of its diphasic effect as a scavenger of ROS in low doses (1-15 *μ*M) and as an inductor of ROS in high doses (20-50 *μ*M) [83].

Novaes et al. [55] reported positive effects of treatment with curcumin alone as well as in combination with benznidazole in controlling oxidative stress. Mice infected with *T. cruzi* Y strain and treated with curcumin for 20 days showed reduced MDA and PCN levels in cardiac and liver tissues compared to that noted in infected/untreated controls. When used in combination with benznidazole, curcumin offered a greater reduction in MDA and PCN levels in cardiac tissue [55]. Contrarily, Nagajyothi et al. [84] showed in mice infected with the Brazil strain of *T. cruzi* that curcumin treatment for 35 days during the acute phase resulted in a decline in the expression levels of antioxidants including SOD, CAT, and peroxidases, though curcumin was beneficial in controlling the parasite burden.

### 3.7. Astaxanthin (ASTX)

ASTX (3,3′-dihydroxy-*β*,*β*′-carotene-4,4′-dione) is a carotenoid found in plants, microorganisms, and sea creatures. Its benefits as an antioxidant include scavenging of radicals, protection of antioxidant enzymes activities, and inhibition of lipid peroxidation [85]. There was only one study examining the effects of ASTX in Chagas disease. Contreras-Ortiz et al. evaluated *in vitro* and *in vivo* effects of ASTX supplementation with or without the antiparasitic drug nifurtimox. Mice treated with ASTX (10 mg/kg/day, orally) for 60 days showed no benefits in controlling MDA levels, irrespective of whether ASTX was given alone or in combination with nifurtimox. The use of ASTX was not even recommended due to an observed increase in MDA levels during the acute phase of *T. cruzi* infection.

### 3.8. Desferrioxamine (DFX)

DFX is an iron chelating agent that has been reported to reduce the formation of free hydroxyl radicals [86], increase the levels of antioxidant enzymes like GPx and SOD [87], and prevent lipid peroxidation with enhanced efficacy when used in combination with other antioxidants (e.g., melatonin) [88]. Francisco et al. [89] have assessed the antioxidant effects of DFX in an experimental model of acute CD. Surprisingly, the authors noted the hepatic and serum levels of thiobarbituric acid reactive species (TBARS) and nitrate/nitrite were increased in infected mice after 21 days of treatment with DFX. This was despite the finding that SOD activity was enhanced throughout the acute phase in DFX-treated infected mice. We must note that SOD activity is increased in the heart and liver of mice acutely infected with *T. cruzi* (i.e., during 8-21 dpi) after which the antioxidant capacity is diminished [32]. Thus, the observed increase in SOD activity in acutely infected mice may not be DFX-dependent [89]. Thus, it might be of interest to evaluate DFX effects during the chronic Chagas disease phase when antioxidant status is usually compromised.

### 3.9. Phytotherapy Compounds

The antioxidant potential of phenolic compounds (flavonoids) in the chronic phase was evaluated using the *Morus nigra* plant extract [90]. Unlike the other studies discussed above, the plant extract was not composed of a specific purified phenolic compound. Nevertheless, the authors determined that the total flavonoid concentration was 361.83 *μ*g/ml of the plant extract. Mice were treated daily with 25 *μ*l, 50 *μ*l, or 75 *μ*l of *M. nigra* extract corresponding to 9 *μ*g, 18 *μ*g, and 27 *μ*g, respectively, of flavonoids for 180 days. However, authors noted no benefits of either of the concentrations of *M. nigra* extracts in enhancing the antioxidant ability or reducing the lipid peroxidation products in infected or uninfected animals.

### 3.10. Carvedilol (Cv)

Cv is a nonselective third-generation *β*-blocker. Although Cv is not an antioxidant, it has been shown to possess both ROS-scavenging and ROS-suppressive effects [91] and offer protection against lipid and protein oxidation mediated by the binding to Fe (III) and Cu (II) transition metals [92]. Further, Cv has been found to decrease serum lipid peroxidation and, thus, exert antioxidant effects [93]. Horta et al. [94] treated the acutely infected mice with 25 mg/kg/day carvedilol for 23 days and showed that in spite of lowered TBARS content in treated mice, Cv was not efficacious in decreasing the protein carbonylation or in enhancing the expression or activities of the SOD and CAT antioxidant enzymes. Codelivery of Cv with benznidazole also showed no benefits compared to what was noted in infected mice treated with benznidazole alone. Authors concluded that Cv is not useful in CD, and it might cause higher inflammation and lower survival rate in infected host.

## 4. Therapeutic Antioxidant Effects in Human Chagas Patients

Chagas cardiomyopathy is the most common and overwhelming manifestation in the chronic phase of *T. cruzi* infection. It is suggested that heart disease occurs by irreversible tissue damage associated with oxidative stress rather than a direct continued action of the parasite [95]. Oxidative stress triggers the immunological responses related to the physiological disturbance and progression of chronic Chagas cardiomyopathy [39]. Thus, antioxidants have emerged as a potential therapeutic option for arresting the tissue injuries in the chronic phase of Chagas disease. Typically, serum and red blood oxidative stress markers are evaluated as an evidence of antioxidant effects. The studies focused on testing the antioxidants' efficacy in Chagas patients are summarized in Table 2 and discussed below.

A research group at the Universidade Federal de Santa Catarina, Brazil, first reported in year 2007 their data obtained from Chagas patients with different stages of chronic heart disease, classified according to modified Los Andes clinical-hemodynamic classification in groups IA, 1B, II, and III (*n* = 10 per group) [39]. Several antioxidant and oxidant biomarkers were measured, and the authors noted that the intraerythrocytic GSH concentrations were decreased in correlation with progression of the disease. This study was followed up by treatment of the same patients' cohort with vitE (800 UI/day) and vitC (500 mg/day) for six months. Macao et al. [96] noted that the vitE/vitC supplementation decreased plasma levels of TBARS and PCN and increased red cell levels of GSH content and GPx and CAT activities in early-stage patients, but this treatment was not sufficient to attenuate oxidative insult in late stage patients.

In another study, Ribeiro et al. [97] measured biomarkers of oxidative stress in Chagas patients (classified as above) before and after two-month treatment with benznidazole and subsequent supplementation with vitE/vitC for six months. Authors noted that BZN treatment enhanced the antioxidants (SOD, CAT, GPx, and GST) as well as protein carbonyls in all groups except group III, and after antioxidant supplementation, the activities of antioxidants as well as biomarkers of oxidative insult (PCN, TBARS, NO, GSH, etc.) were decreased. The authors concluded that BZN treatment promoted an oxidative insult while the antioxidant supplementation was able to attenuate this effect by increasing vitamin E levels, decreasing PCN and TBARS levels and inhibiting SOD, GPx, and glutathione reductase (GR) activities as well as inflammatory markers, mainly in patients with the less severe form of cardiac involvement. Further, Barbosa et al. [98] noted that patients with advanced heart disease exhibited a decline in premature ventricular contraction after BZN and vitE/vitC treatment.

A similar patient cohort as discussed above was treated with carvedilol and vitE/vitC supplements for six months and followed to determine if concomitant use of carvedilol will enhance the antioxidant effects of vitE and vitC [99, 100]. Budni et al. [99, 100] noted significant decline in oxidative stress levels in patients that received carvedilol alone or in combination with vitE/vitC, the maximal benefits being noted in patients that received Cv and vitE/vitC together, thus suggesting the possibility of synergism between these compounds. Again, maximal benefits of Cv and vitE/vitC in attenuating the systemic oxidative stress were noted in patients with the less severe form of heart Chagas disease.

Other elements with antioxidant properties such as selenium have also been investigated as an adjuvant therapy in CD [101]. Nonetheless, these studies were focused on controlling the heart dysfunction and none evaluated the effects of the treatment on oxidative stress markers.

## 5. Outlook and Future Perspectives

The antioxidant adjuvant therapy is anticipated to decrease ROS in the host tissues; however, we have also shown here that vitC and vitE in the Chagasic host exert a synergistic trypanocidal effect as they cause an increase in reactive species that trigger redox imbalance and parasite death [102]. New studies, however, are needed to determine if vitC/vitE treatments eventually result in improved heart function in Chagas disease.

Our review highlights the reduction of plasmatic and tissue-lipid peroxidation levels as a major benefit provided by antioxidant therapies (Table 3). Curcumin, resveratrol, and Cv as well as metformin and Tempol showed this benefit in preclinical studies, although only Cv has been evaluated in Chagasic patients. Other antioxidants showed detrimental or no positive effects in Chagas disease. However, we must note that many of the antioxidant therapies have only been evaluated once and others have not reproduced the published studies. Thus, the evidence on whether antioxidant supplementation can reduce oxidative stress in Chagasic patients is insufficient right now and further studies are necessary to confirm the initial results.

The benefits of antioxidants seem greater when they are used in combination with or after antiparasitic drug administration, causing a reduction in plasmatic and cardiac lipid peroxidation. Some of the antioxidants enhanced parasite clearance as well. Thus, it is recommended that future studies should focus on evaluating antioxidant compounds in conjunction with antiparasitic therapies.

There are multiple antioxidants evaluated in CD, and some of them have shown promising results and potential benefits in the improvement of oxidative stress parameters in affected tissues. Studies on changes in oxidative stress parameters after antioxidant supplementation, alone or in combination with antiparasitic drugs, offer new hope for halting the progression of cardiac dysfunction in Chagas heart disease. This possibility must be investigated both in the acute phase and later during the chronic phase and focused on the oxidative stress imbalance. While evidence supports the association of oxidative stress with the development of cardiac alterations and that adjuvant therapy with some antioxidants modifies tissue oxidative stress parameters, the relation between antioxidants and the development or progression of tissue damage in the main organs affected by CD still needs to be further investigated. In the same way, there are no previous studies or comprehensive assessment of the signaling pathways involved in oxidative stress reduction related to antioxidant supplementation in CD. It is also necessary to explore a specific and appropriate supplementation time or dose-dependent effects that show an advantage to control oxidative stress and consequent cardiac damage and left ventricular dysfunction in Chagas disease.

## Figures and Tables

**Table 1:** Evidence supporting antioxidant use in Chagas disease as revealed by research in animal models.

Reference #	Author, year	Experimental model	Age in weeks	*T. cruzi* strain (trypomastigotes challenge dose)	Treatment	Antioxidant/oxidative stress markers	Tissue samples
[94]	Horta et al., 2018	C57BL/6 male mice	8-10	Colombian (50)	25 mg/kg/day carvedilol ± 100 mg/kg/day benznidazole for 23 days by gavage	SOD and CAT activity, TBARS, protein carbonyls	Heart
[52]	Castanheira et al., 2018	Swiss SWR/J male mice	3.5	QM2 (5 × 10^4^)	500 mg/day vitamin C for 60 days in drinking water	FRAP, GSH, GST, plasma sulfhydryl (SH) group, nitrate/nitrite	Plasma, heart, colon, skeletal muscle
[58]	Providello et al., 2018	Swiss SWR/J male mice	6	Y (1 × 10^4^)	7.14 mg/kg/day vitamin C ± 100 mg/kg benznidazole for 15 days by gavage	TBARS ROS	Heart, macrophages
[56]	Contreras-Ortiz et al., 2017	BALB/c female mice	4-6	Ninoa (10)	10 mg/kg/day astaxanthin ± 100 mg/kg/day nifurtimox for 60 days orally	MDA	Heart, spleen, blood
[90]	Montenote et al., 2017	Swiss SWR/J male mice	3	QM2 (5 × 10^4^)	20% blackberry plant extract (25-75 *μ*l/day) 180 days orally	TBARS, FRAP, GSH, and sulfhydryl groups	Plasma
[53]	Tieghi et al., 2017	Swiss SWR/J male mice	3	QM2 (5 × 10^4^)	500 mg/day vitamin C and 800 IU/day vitamin E (individually and in combination) for 60 days or 120 days	FRAP, GSH, TBARS	Plasma, heart, colon, and skeletal muscle
[48]	Novaes et al., 2017	Swiss SWR/J male mice	52	Y (2 × 10^3^)	500 mg/day vitamin C/800 UI/day vitamin E for 15 days orally	TBARS, PCN, catalase, GST and SOD activities, nitrite/nitrate, 8-OHdG	Heart
[69]	Vilar-Pereira et al., 2016	BALB/c male & female mice	5-7	Colombian (2 × 10^2^)	15 mg/kg *trans*-resveratrol (i.p.) or 40 mg/kg resveratrol, 500 mg/kg metformin, 100 mg/kg/Tempol, or 25 mg/kg benznidazole for 30 days (per orally)	TBARS	Heart
[55]	Novaes et al., 2016	SWR/J female mice	8-12	Y (2 × 10^3^)	Curcumin (C) ± benznidazole (B) for 20 days by gavage, C100 (±B50-B100), B50-B100 only (mg/kg/day)	MDA and PCN	Heart, liver
[54]	Brazao et al., 2015	Wistar male rats	NR	Y (1 × 10^5^)	5 mg/kg melatonin/day for 60 days orally	Nitrite production in macrophages; TBARS in plasma	Plasma, spleen
[60]	Marim et al., 2015	SWR/J male mice	3	QM1 (5 × 10^4^)	10 *μ*l vitamin C (D60 mg or D500 mg) per day for 60 days or 180 days orally	TBARS, total peroxide, GSH	Plasma, heart, colon, skeletal muscle
[84]	Nagajyothi et al., 2012	CD1 mice	6-8	Brazil (5 × 10^4^)	100 mg/kg/day curcumin for 35 days orally	mRNA levels of enzymes/proteins	Heart
[64]	Wen and Garg, 2012	Sprague Dawley rats	4–5	Sylvio ×10 (5 × 10^4^)	1.3 mM PBN and/or 0.7 mM benznidazole for three weeks in drinking water	PCN	Heart, heart mitochondria
[51]	Wen et al., 2010	Sprague Dawley rats	4–5	Sylvio ×10 (5 × 10^4^)	1.3 mM PBN and/or 0.7 mM benznidazole for three weeks in drinking water	ROS, TBARS	Heart, heart mitochondria
[89]	Francisco et al., 2010	SWR/J male mice	4	Y (5 × 10^2^)	5 mg/50 *μ*l/day desferrioxamine (i.p.) 14 days prior to infection and for 21 days i.p.	GSH, TBARS, PCN, nitrate/nitrite	Serum, liver
[50]	Wen et al., 2006	C57BL/6 mice	6-8	Sylvio ×10 (1 × 10^4^)	50 mg/kg PBN (i.p.) on alternate days for three weeks	Respiratory complex activities, MDA, GSH, ATP, H_2_O_2_	Heart, heart mitochondria

Cv: carvedilol; SOD: superoxide dismutase; CAT: catalase; MDA: malondialdehyde; GSH: glutathione; GST: glutathione S-transferase; FRAP: plasma antioxidant capacity; TBARS: thiobarbituric acid reactive substances; PCN: protein carbonyl levels; PBN: phenyl-alfa-tert-butyl nitrone; B100: 100 mg/kg of body weight benznidazole; B50: 50 mg/kg benznidazole; C100: 100 mg/kg curcumin; dpi: days postinfection.

**Table 2:** Evidence supporting antioxidant use in Chagas disease as revealed by research in humans.

Ref.	Author	Patients with chronic Chagas heart disease (ChD)	Antioxidant	Treatment	Oxidative stress markers examined
[96]	Macao et al., 2007	*n* = 41	Vitamin E (800 UI/day) and vitamin C (500 mg/day)	Benznidazole (5 mg/kg) for 2 months, vitamins for 6 months	SOD, CAT, GPx, GST, GR, GSH, TBARS, PCN, NO, MPO, GGT
[98]	Barbosa et al., 2014	*n* = 41, 41% males, 31-67 years old	Vitamin E (800 UI/day) and vitamin C (500 mg/day)	Benznidazole (5 mg/kg/day) for 2 months, vitamins for 6 months	SOD, CAT, GPx, GST, GR, GSH, TBARS, PCN
[97]	Ribeiro et al., 2010	*n* = 41	Vitamin E (800 UI/d) and vitamin C (500 mg/d)	Benznidazole (5 mg/kg) for 2 months, vitamins for 6 months	SOD, CAT, GPx, GST, GR, GSH, TBARS, PCN, NO, MPO, ADA
[99]	Budni et al., 2012	*n* = 42, 21-70 years old	Carvedilol (37.5 mg/day)	6 months	SOD, CAT, GPx, GST, GR, GSH, TBARS, PCN, NO, MPO, ADA
[100]	Budni et al., 2013	*n* = 42, 21-70 years old	Carvedilol (37.5 mg/day) and vitamin E as above	6 months	SOD, CAT, GPx, GST, GR, GSH, TBARS, PCN, NO, MPO, ADA

Abbreviations: SOD: superoxide dismutase; CAT: catalase; MDA: malondialdehyde; GSH: glutathione; GST: glutathione S-transferase; GR: glutathione reductase; GPx: glutathione peroxidase; TBARS: thiobarbituric acid reactive substances; PCN: protein carbonyl levels; NO: nitric oxide; MPO: myeloperoxidase; GGT: gamma-glutamyl transferase; ADA: adenosine deaminase.

**Table 3:** Summarized presentation of oxidative stress markers and effects of antioxidant supplements evaluated in *T. cruzi* infection and Chagas disease.

Antioxidant	Oxidative stress marker						
	Mitochondrial function	NO production	Lipid peroxidation	PCN	Glutathione	SOD	CAT
PBN	+						
Melatonin		X	+				
Curcumin			+	+		D	D
Resveratrol			+			D	
ASTX			X				
DFX		X	X			I	
Flavonoids (*Morus nigra*)			X				
Vitamins C or vitamin E			X			X	X
Vitamins C/vitamin E			=		+		
Carvedilol			=	X		X_Α_, D_H_	X

+: studies show statistically significant changes of diminishing oxidative stress markers; X: studies show statistically significant detrimental results in oxidative stress markers; =: no changes in measurements in oxidative stress markers; I or D: studies show increase or decrease in antioxidant enzyme levels after antioxidant exposure, respectively; Α: animal models; H: human patients; NO: nitric oxide; PCN: protein carbonylation; glutathione: GSH, GPx, or GST; SOD: superoxide dismutase; CAT: catalase. PBN: phenyl-alfa-tert-butyl nitrone.

## Abbreviations

CD: Chagas disease
Cv: Carvedilol
DFX: Desferrioxamine
eNOS: Endothelial nitric oxide synthase
4-HNE: 4-Hydroxynonenal
iNOS: Inducible nitric oxide synthase
MDA: Malondialdehyde
mtROS: Mitochondrial ROS
NO: Nitric oxide
NOX2: NADPH oxidase 2
PBN: Phenyl-*α*-tert-butyl nitrone
PCN: Protein carbonyl
RNS: Reactive nitrogen species
ROS: Reactive oxygen species
SOD: Superoxide dismutase
TBARS: Thiobarbituric acid reactive species
*T. cruzi*: *Trypanosoma cruzi*.

## Additional Points

*Highlights*. *Trypanosoma cruzi* infection leads to chronic Chagas cardiomyopathy. A positive correlation between peripheral and cardiac levels of oxidative and nitrosative stress and the severity of heart disease is reported in experimental models and human patients of Chagas disease. A compromised antioxidant defense of the host contributed to myocardial oxidative damage presented by increased lipid peroxidation, protein carbonyls, and DNA adducts. The drugs used to treat *T. cruzi* infection, nifurtimox and benznidazole, induce increased ROS and DNA damage in host tissues. Combinatorial antiparasitic therapies with antioxidants have potential clinical relevance in arresting oxidative injuries. It is necessary to assess the effects of antioxidant supplementation in various disease phases to offer a unifying treatment recommendation.
